# A Semi-Physical Platform for Guidance and Formations of Fixed-Wing Unmanned Aerial Vehicles

**DOI:** 10.3390/s20041136

**Published:** 2020-02-19

**Authors:** Jun Yang, Arun Geo Thomas, Satish Singh, Simone Baldi, Ximan Wang

**Affiliations:** 1 Systems Engineering Research Institute, China State Shipbuilding Corporation, Beijing 100094, China; 2 Delft Center for Systems and Control, Delft University of Technology, 2626CD Delft, The Netherlandsx.wang-15@tudelft.nl (X.W.); 3 School of CyberScience and Engineering, Southeast University, Nanjing 211189, China; 4 School of Mathematics, Southeast University, Nanjing 211189, China

**Keywords:** Unmanned Aerial Vehicles (UAVs), fixed-wing UAVs, smart sensor systems, guidance law, formation control, cyber-physical systems

## Abstract

Unmanned Aerial Vehicles (UAVs) have multi-domain applications, fixed-wing UAVs being a widely used class. Despite the ongoing research on the topics of guidance and formation control of fixed-wing UAVs, little progress is known on implementation of semi-physical validation platforms (software-in-the-loop or hardware-in-the-loop) for such complex autonomous systems. A semi-physical simulation platform should capture not only the physical aspects of UAV dynamics, but also the cybernetics aspects such as the autopilot and the communication layers connecting the different components. Such a cyber-physical integration would allow validation of guidance and formation control algorithms in the presence of uncertainties, unmodelled dynamics, low-level control loops, communication protocols and unreliable communication: These aspects are often neglected in the design of guidance and formation control laws for fixed-wing UAVs. This paper describes the development of a semi-physical platform for multi-fixed wing UAVs where all the aforementioned points are carefully integrated. The environment adopts Raspberry Pi’s programmed in C++, which can be interfaced to standard autopilots (PX4) as a companion computer. Simulations are done in a distributed setting with a server program designed for the purpose of routing data between nodes, handling the user inputs and configurations of the UAVs. Gazebo-ROS is used as a 3D visualization tool.

## 1. Introduction

The availability of low-cost sensors, electronics, and air-frames has promoted a significant interest in Unmanned Aerial Vehicles (UAVs) among aircraft hobbyists, academic researchers, and industries [1,2]. Among the different families of UAVs, the fixed-wing type is generally deployed when extensive areas are to be covered in a short time. In fact, this type of UAV can fly with considerable speeds and with efficient aerodynamics [3,4]. While fixed-wing UAVs were initially studied especially by military and government organizations, nowadays commercial and civil applications of fixed-wing UAVs are envisaged, and one popular example is carrying payloads [5,6,7]. This and other applications require the UAV to autonomously follow a predefined path at a prescribed height [8,9]. Flying formations of multiple fixed-wing UAVs has also become a significant topic of research due to numerous defense and commercial applications including reconnaissance and surveillance missions, coordinated attacks, flying into high-risk areas, livestock monitoring, wildfire mapping, etc. [10,11].

Despite the increasing interest in fixed-wing UAVs, few simulation environments are available that can simulate all the complex architecture of such an autonomous system. Often, Matlab/Simulink environments are used to simulate and visualize UAVs, which require making significant simplifications in design and simulations [12,13,14]. For example, in recent survey papers on path planning for UAVs [15,16], all comparisons among the different algorithms (vector-field, carrot-chasing, nonlinear guidance, pure pursuit with line-of-sight, linear quadratic regulation) are made assuming a point mass kinematic model for the UAV. Similarly, most designs for the guidance laws are based on considering simplified first-order course dynamics [17,18,19,20]; however, the UAV course dynamics are the result of the low-level control layer (e.g., roll and pitch control loops) implemented in the autopilot layer on board of any UAV [21]. The low-level autopilot layer results in more complex course dynamics than first order. Very often such low-level autopilot layer is simply neglected, leading to simplified first-order course dynamics and lack of realism. In general, it is cumbersome to interface computing environments as Matlab/Simulink with the hardware components actually used on board UAVs. As a matter of fact, the autopilot layer and the communication layer are the most challenging layers to include in a simulation environment. The autopilot layer contains a huge suite of algorithms that are cumbersome to replicate in Matlab/Simulink (software-in-the-loop configuration); at the same time, Matlab/Simulink cannot communicate directly with the autopilot hardware (hardware-in-the-loop configuration) and ad-hoc protocols to allow such communication should be designed. Similarly, it is quite challenging to replicate in Matlab/Simulink all the features of wireless communication (software-in-the-loop configuration); at the same time, hardware-in-the-loop configuration requires again ad-hoc protocols to allow communication among different layers [22,23,24]. Neglecting the autopilot layer and the communication layer can (to some extent) be acceptable for quadrotor type of UAVs, since they have to fly at relatively low velocity [25,26]; however, fixed-wing UAVs demand a much more realistic platform in order to evaluate their performance at high speed, high altitudes and in demanding manuevers affecting the aerodynamics. Based on literature and on the authors’ experience, the lack of a realistic simulation platform for fixed-wing UAVs prevents assessing the following key points:

1. The actual performance of guidance methods considerably depends on the fidelity of the UAV model used for design. Parametric uncertainties and unmodelled dynamics will certainly appear in the UAV structure and cannot be captured by point mass kinematic models [27].
2. The actual path-following performance does not depends only on the commanded course angle. The autopilot in charge of regulating roll, pitch and altitude (rudder/wing/aileron actuators) crucially contributes to the final performance [28]. The integration of the autopilot layer should constitute an important part in semi-physical simulations towards real flight testing.
3. As the guidance algorithm cannot always sit in the autopilot (due to intensive computation requirements), it should be implemented on extra hardware companion computer. Such extra hardware should be embedded with wireless communication capabilities: Therefore, a proper server program handling the data exchange for UAV formation algorithms should be put in place.

This work will present a semi-physical simulation platform for multi fixed-wing UAVs with the capability of capturing several physical dynamics (aerodynamics, wind environment), as well as several complex dynamics such as:

- Communication protocols among UAVs and between UAVs and ground station, with possibility to simulate communication losses across links;
- Path manager dynamics, mainly dividing the mission into primitives of straight lines/orbits, and switching from orbit to straight line missions (and viceversa);
- Simulate formations of fixed-wing UAVs and simulate transitions from one formation to another while being airborne.

The proposed environment adopts Raspberry Pi’s that run guidance and formation control algorithms programmed in C++: Such Raspberry Pi’s can be interfaced to standard autopilots (PX4) as a companion computer. Simulations are done in a distributed setting with a server program designed for the purpose of handling the user inputs and configurations of the UAVs. Gazebo-ROS is used as a 3D visualization tool.

The article is organized as follows: Section 2 introduces the simulator components with emphasis on the Gazebo-ROS environment. Section 3 describes the communication architecture, in particular a server program to handle wireless communication. Section 4 and Section 5 give an overview of the UAV physics and of the control architecture, especially for the formation control part. Simulations are in Section 6 with concluding remarks in Section 7.

## 2. Overview of the Simulator Components

**Figure 1:**
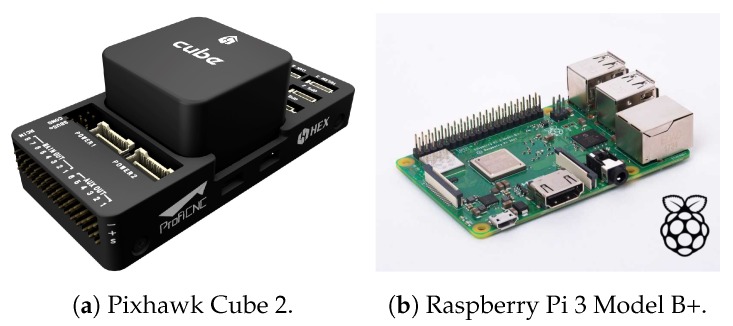
Hardware to implement guidance and formation control algorithms.

UAVs need onboard flight controllers which enable stable flight and communication link to a ground station. The onboard flight controllers, also called autopilot, are realised using both hardware and software. The system under consideration uses Pixhawk cube (PX4-(v2)/cube) hardware shown in Figure 1a. Pixhawk cube (Dronecode Project, Inc., a Linux Foundation Collaborative Project. Linux Foundation is a registered trademark of The Linux Foundation. Linux is a registered trademark of Linus Torvalds) has a 32 bit STM32F427 Cortex-M4F core with servo outputs that can be interfaced with actuators on the UAVs. The hardware board also has inertial measurement units for measuring inertial parameters. The Pixhawk cube runs the NuttX Operating System to supply basic real-time operating system features to the autopilot software. The autopilot software contains the codes to enable multiple features to aid UAV flight control and navigation: Pitch control, roll control, velocity and altitude control, state estimation (such features are explained in this work only briefly for compactness) [29]. Two established software stacks for UAV autopilot are Ardupilot and PX4, both supported by Pixhawk cube. In this work we will make use of PX4.

For formation control of a swarm of UAVs, each UAV needs to transmit and receive information with a ground station (containing the formation control algorithms and desired missions), and/or to transmit and receive information with the neighbouring UAVs (in case the formation control algorithms are implemented on the UAVs themselves). Thus, the UAVs need modules enabling inter-node communications: With the data received, UAVs need to execute formation control algorithms. The computational power of Pixhawk boards is only meant for enabling basic UAV operations and does not suffice for executing such algorithms. Pixhawk together with the autopilot software supports the so-called Offboard Mode, where the systems can receive commands from a companion computer with higher computational power. In this work, Raspberry Pi ( Raspberry Pi Foundation, UK registered charity 1129409, Cambridge, UK), a series of small and cheap single-board computers widely used in embedded applications, is adopted to this purpose. In particular, Raspberry Pi 3 Model B+ (1.4 GHz 64-bit quad-core processor with dual-band wireless LAN, cf. Figure 1b) is employed as companion computer. The wireless LAN can be used to establish a communication mechanism for data exchange between the nodes.

**Figure 2:**
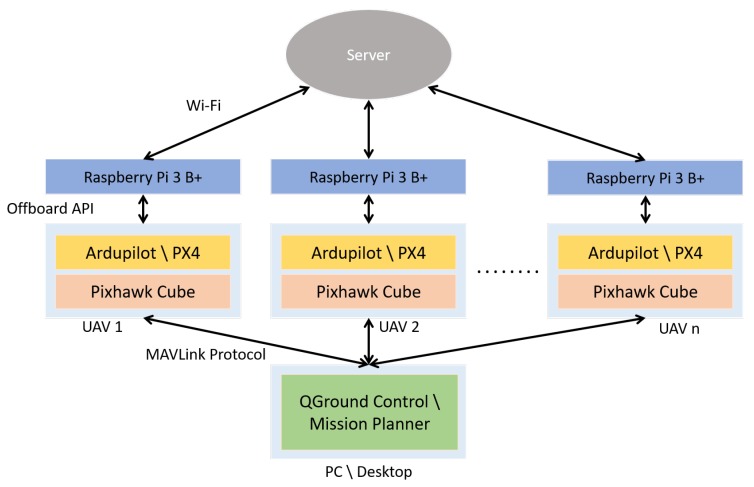
System architecture for Unmanned Aerial Vehicle (UAV) formation control.

The user can configure or interact with each individual UAV using a ground station software: QGroundControl and Mission Planner are two well-known software suites. In this work we will adopt QGroundControl. The autopilot stack and ground station communicate using MAVLink protocol. The proposed complete system architecture for UAV formation control is shown in Figure 2. For visualization of the UAVs, among several options (Unity 3D, X-plane and jMAVSim, etc.), Gazebo with Robot Operating System (ROS) has been adopted in this work. Three types of semi-physical simulations are enabled by Gazebo: It can be used as software-in-the-loop (SITL), or hardware-in-the-loop (HITL) or Simulation-In-Hardware (SIH) simulator for a single UAV. These three types of semi-physical simulations are as follows:

- Software-in-the-loop (SITL) simulation: It is performed when all the required components (UAV dynamics, autopilot, guidance and formation control) sit in the same machine. Communication overhead is reduced; however, because the performance of a computer is not the same as that of a microcontroller used during real flight, the simulated performance may differ from the actual performance in real scenarios.
- Hardware-in-the-loop (HITL) simulation: It is performed using a real flight microcontroller board attached to the simulated environment with the model of the UAV. This simulation is useful to show how a microcontroller-based system responds in real time to virtual stimula.
- Simulation-in-hardware (SIH) simulation: It is an alternative to HITL in which everything (UAV dynamics, autopilot, guidance and formation control) runs on embedded hardware. A machine is only used to display the virtual UAV. As compared to HITL, SIH avoids the bidirectional connection to the machine, thus reducing the round trip delay introduced by simulation in a remote environment. Refer to https://dev.px4.io/master/en/simulation/simulation-in-hardware.html for details on SIH mode.

It must be underlined that one possible way to achieve HITL is to connect Pixhawk through USB cable to the Ubuntu machine running Gazebo (without companion computer): In this configuration the autopilot stack is uploaded both to the hardware itself and to the host machine. Gazebo runs the physical model of the UAV and sends sensor outputs to PX4 via USB. PX4 acknowledges the data and generates the control action to close the loop with the simulator. This works fine for a single UAV. However, for multiple UAVs, the autopilot stack is uploaded to the multiple PX4 boards which are then latched to the Gazebo machine through USB. Multiple ROS nodes are generated to publish and subscribe the data and command for each vehicle. Unfortunately, the communication burden associated with such an architecture causes a communication loss along the USB link of more than 50% even with only two UAVs. In the following subsections we will explore SITL and an alternative HITL with companion computer (which helps addressing the loss of data problem). The design of a SIH environment can be the object of future work.

Alternatives proposed in literature rely on connecting one UAV hardware per computer and use a distributed simulation approach to integrate all UAVs in a unique environment [30,31]. This option is not explored here as the interest is on using a unique computer.

### 2.1. SITL Environment

For software-in-the-loop simulation, Gazebo is used as simulator, PX4-(v2)/cube as an autopilot stack, and QGroundControl as a ground control station. For multiple UAVs, MAVROS is used: MAVROS is a package that utilizes MAVlink communication protocol to provide communication driver for various autopilots. MAVLink is a communication protocol specifically developed for aerial robots or drones. It is a very light weight messaging protocol, with hybrid publish-subscribe and point-to-point design pattern: Data streams are sent/published as topics while configuration sub-protocols such as the mission protocol or parameter protocol which are point-to-point with retransmission.

**Figure 3:**
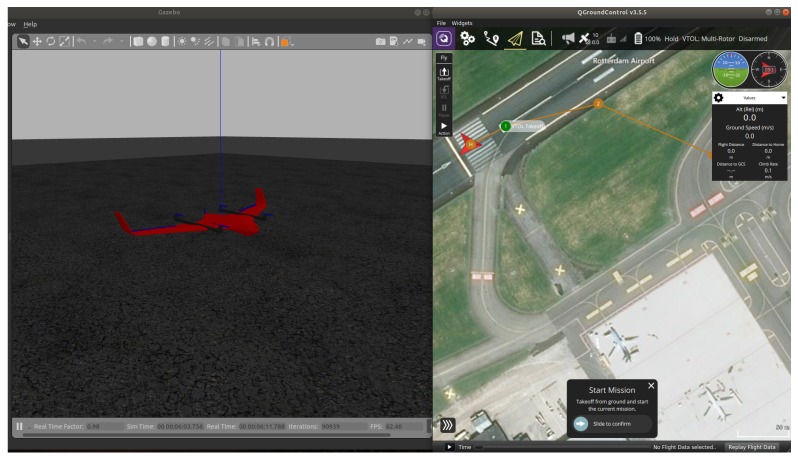
Gazebo with ground control station.

For the purpose of this project, a vertical take off and landing (VTOL) UAV is used, which combines the feature of both quadrotors and fixed-wing UAVs. The path was planned using waypoints such as straight line path and loiter points as shown in Figure 3.

**Figure 4:**
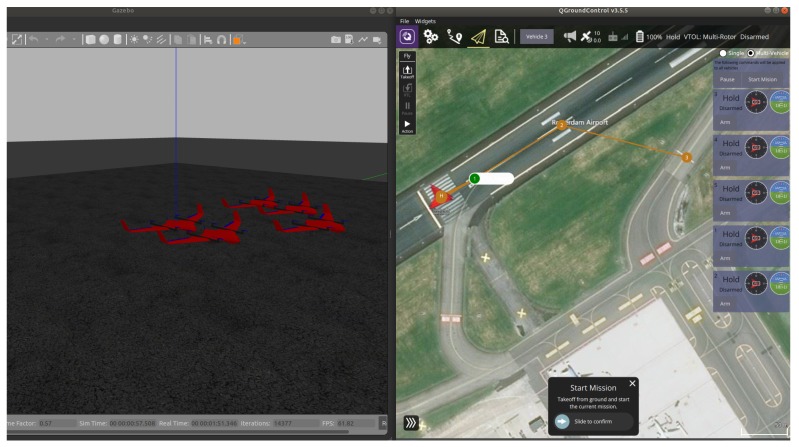
Launching five UAVs.

**Figure 5:**
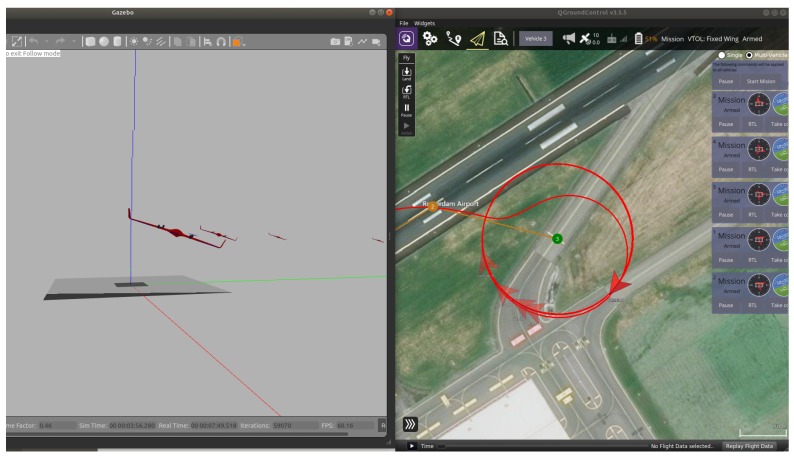
UAVs during flight and loitering.

To handle multiple UAVs (cf. Figure 4), a MAVlink message is defined with argument (mavlink_udp_port) inside the SDF file of Gazebo to handle the communication with PX4. The UDP port is configured in the PX4 node and set in the start up file in the application side (SITL_UDP_PRT) parameter. MAVlink bridges the same UDP port defined in (mavlink_udp_port) to communicate with the autopilot. The Gazebo launch file must contain the vehicle type and vehicle ID: For consistency, every vehicle to be launched with the MAV_SYS_ID in the start-up file must match with the vehicle ID inside launch file. Figure 5 shows the the ground control station and Gazebo with the UAVs during flight.

### 2.2. HITL Environment

**Figure 6:**
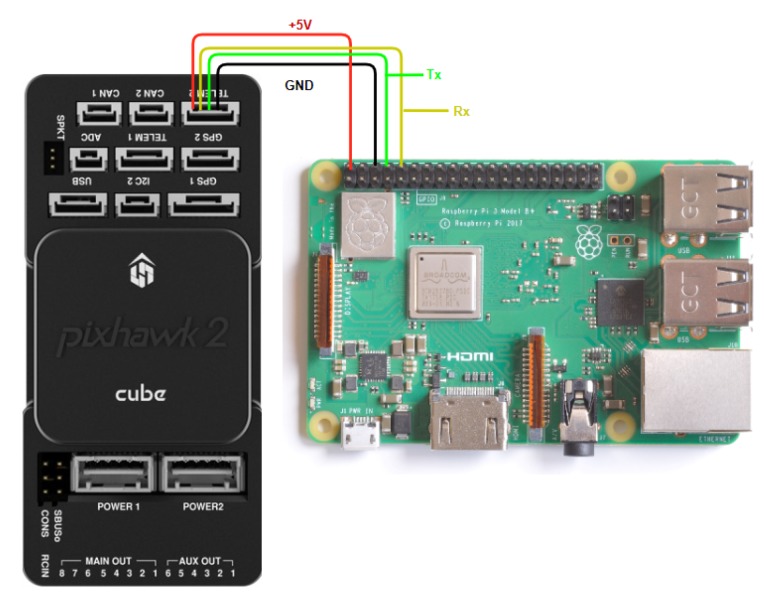
Companion computer (RPI 3 b+) connection to autopilot (Pixhawk2).

The following configuration with Raspberry Pi as a companion computer along with PX4 board allows to tackle the problem of communication losses with multiple UAVs: The idea is to connect Raspberry Pi and Pixhawk using the MAVlink protocol over serial connection, see Figure 6. The serial port on a PX4 board can be fully configured from QGroundControl via the parameters:

- MAV_COMP_ID = 2: MAVlink component ID.
- MAV_2_CONFIG = TELEM 2: Serial Configuration for MAVLink (instance 2) This parameter configures the port for serial communication using MAVlink. Here, it configures the serial port to run MAVlink on port 102 (for TELEM 2).
- MAV_2_MODE = 2: Option 2 is used here for Onboard standard set of messages. This MAVink mode defines the set of streamed messages and their sending rates for a companion computer.
- MAV_2_RATE = 921600 baud: This is a parameter for configuring the maximum sending rate. The sending rate of the messages from each stream automatically lowers down if the configured stream exceed the maximum defined rate.
- MAV_2_FORWARD = True: If mode is enabled and either the point of reference is not the autopilot or it is broadcast, this configuration forwards the incoming MAVlink messages to other MAVlink ports. With help of this, QGroundControl is able to talk to the companion computer that is connected to autopilot via MAVlink protocol.

**Figure 7:**
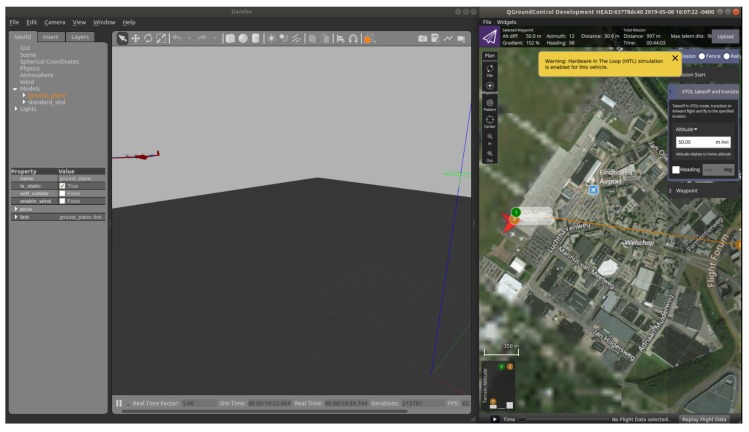
Hardware-in-the-loop (HITL) simulation with companion computer.

For the quadrotor mode of VTOL, the HITL simulation is always successful as shown in Figure 7; however, the transition from quadrotor to fixed-wing is more delicate and still ongoing, due to fact that the fixed-wing libraries on the autopilot stack are not as developed as the quadrotor libraries [32]. In fact, it has to be noted that there is no mode defined on the PX4 autopilot stack that allows HITL of fixed-wing UAV [33]. Therefore, the authors had to develop their own flight mode for fixed-wing mode, which is still under development and not definite:these aspects make interfacing of the companion computer with with autopilot more challenging. Fixing these issues will be explored in future work. Another interesting option worth exploring in the future is the integration of Raspberry Pi via the Pulse Width Modulation (PWM) channel (the same one used for remote control (RC)). Thus, the Raspberry Pi could control the UAV sending similar commands sent by RC [34].

## 3. Communication Architecture

The proposed complete system architecture for UAV formation control is as in Figure 2. In order to implement the formation algorithm, all Raspberry Pi’s should be connected to a server program through Wi-Fi. The server program has two primary functions: (1) Receive user input for the configuration for formation flying and configure the UAVs accordingly, (2) Receive and distribute data between the UAVs. In the following, let us describe the main features of the server program.

### 3.1. Synchronization of Data between Nodes

We follow a server-client architecture for data transfer, operating in a network having only one subnet. The server computer is a computer which runs the server program for distributing data across the Raspberry Pi nodes. The server program can sit in the same machine running Gazebo, or in a different machine. Here we define data Synchronisation as the timed update of data of a node stored in a second node. We use UDP for data transfer and we define a higher level protocol which operates above the Transport layer, customized for the application of formation control.

On the sender node side, the message data is wrapped in an object of class Packet using the helper functions. The packet is serialized using the function GetByteStream and resultant byte stream is sent over the network to the receiver node using the IP address of the node. In order to read out a packet, we need to find the start byte of the packet. If there are missing bytes of a packet, we need to drop the section and start searching for a start byte again. The packet length can vary depending on the size of the message. In order to ensure all these requirements, we introduce a Finite-State Machine based algorithm to readout packets from the data available in the read buffer. The state machine in packet reception algorithm has seven states. The finite states for the packet reception algorithm are:

1. Check Start Byte. The state machine starts in state Check Start Byte. In Check Start Byte, algorithm searches for a read byte in the read buffer. Till it finds a candidate for start byte, state machine continues in the state. Once it spots a candidate, the state machine advances to state Check Message Type.
2. Check Message Type. State Check Message Type checks whether the next byte is a valid candidate for message type. If the byte is a valid candidate, state machine progresses to the state Get Drone ID otherwise falls back to start byte check.
3. Get Drone ID. The state machine in state Get Drone ID uses the next byte, saves it as drone ID and moves to the state of Get Data Length.
4. Get Data Length. State Get Data Length sets the next byte as the length of the data bytes and moves to state Get Message.
5. Get Message. State machine loops in the state Get Message until sufficient data bytes as per the data length is obtained. On completion, state machine advances to state Check CRC0.
6. Check CRC0 and Check CRC1. State Check CRC0 is followed by state Check CRC1. Both states together ensure the integrity of the received packet using the two CRC bytes. In case of CRC check failure, the data bytes are rewound and the state machine starts searching for another start byte from the spot next to where the last start byte was detected. If the CRC check succeeds, there is a valid packet reception, and the state machine starts to read from new bytes in the buffer to process the next packet.

**Figure 8:**
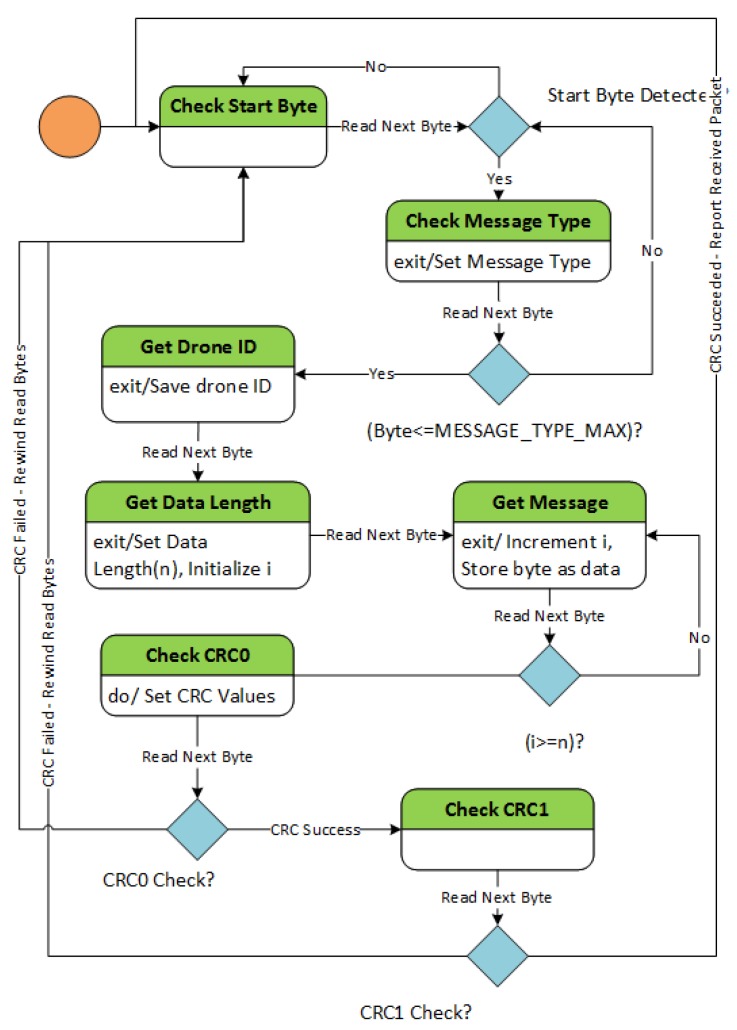
State diagram for packet reception.

The state diagram for the packet reception algorithm is given in Figure 8.

The packet receiver is implemented as class Packet Receiver. The class has functions that can be called with a filled buffer. The function either finds the last packet among a read buffer or uses a handler to process each available packet. If there are not enough bytes to complete a packet read in the buffer, the instance of packet receiver can be retained and can resume reading with the new set of bytes received. The packet receiver internally uses the unconsumed bytes from the previous attempt along with the new bytes.

### 3.2. Server for Data Synchronisation

The Raspberry Pis send data to the server program using the protocol and mechanism introduced in the previous sections. The server needs to process the data packets from the bytes sent by each node and distribute it to other nodes which are in need.

The server program has to serve all the UAV nodes judiciously. The program should not listen and serve one node alone for a long period of time. In order to address this concern and to have enough decoupling, a multi-threaded server design is required.

There are four main tasks for the server, they are:

1. Handle user configurations and inputs.
2. Read bytes from all the nodes sent through the network.
3. Process the read bytes to packets and check integrity.
4. Distribute the received packets to interested nodes.

**Figure 9:**
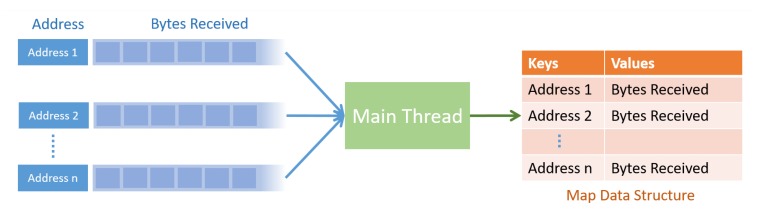
Main thread operation on received bytes.

For compactness and intellectual property agreements, not all details will be disclosed. The main thread continuously reads the data received from the network using the function recevfrom. In every call of the function recevfrom, together with the data, the address of the sender node is obtained. The main thread registers the received data in a map data structure with the address of the sender as the key. The operation of main thread is summarized in Figure 9.

**Figure 10:**
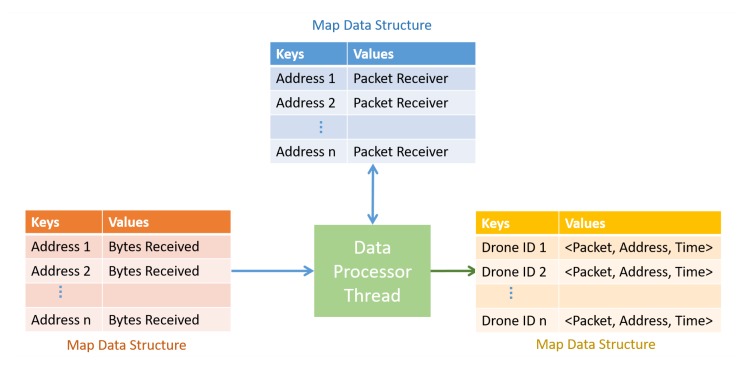
Operation of data processor thread.

Data processor thread, visualized in Figure 10 operates on the map data structure created by the main thread. For every address, an instance of class PacketReceiver is initiated and stored in another map data structure with the address as the key. This helps in resuming from the left out bytes after a processing attempt. The thread uses the instance of class PacketReceiver and processes the bytes to packet. Once the bytes are processed to the packets, we can be sure of the drone ID of the packet. The packets with message type T_DATA are conveniently stored in another map data structure whose keys are drone IDs. The values in the map are tuples consisting of the received packet, the address of drone, and the time of the reception.

One improvement to be explored is related to this issue: While a multi-thread approach on the server-side is enough for equally distributing the time among the devices, it might fail to guarantee real-time performance. For example, if the number of UAV increases, the average waiting time will increase together. A distributed simulation approach as in [30,31] with time-step synchronization could model the more realistic case where multiple devices run in parallel.

## 4. Fixed-Wing UAV Dynamics

In this section, let us quickly describe the underlying dynamics of the fixed-wing UAVs. The interested reader is referred to [35,36] for a more detailed presentation.

The dynamics of a network of fixed-wing UAVs can be described in the Euler–Lagrange framework by

(1) $$D_{i}\left(q_{i}\right)\ddot{q_{i}}+C_{i}\left(q_{i},\dot{q_{i}}\right)\dot{q_{i}}+g_{i}\left(q_{i}\right)=\tau_{i},\;i=\left\{1,\ldots ,N\right\}$$

where the term $D_{i}\left(q_{i}\right)\ddot{q}$ is proportional to the second derivatives of the generalized coordinates, the term $C_{i}\left(q_{i},\dot{q_{i}}\right)\dot{q}$ is the vector of centrifugal/Coriolis forces, proportional to the first derivatives of the generalized coordinates, and the term $g_{i}\left(q_{i}\right)$ is the vector of potential forces. Finally, the term $\tau_{i}$ represents the external force applied to the system: Clearly for a fixed-wing UAV the system is not fully actuated; however, for the sake of simplicity, we will proceed our analysis assuming that the system is fully actuated. Then, in simulations, a control allocator will be put in place to transform the input $\tau_{i}$ into the actual inputs to the system: This will introduce unmodelled dynamics for which robustness of the control will be verified.

**Figure 11:**
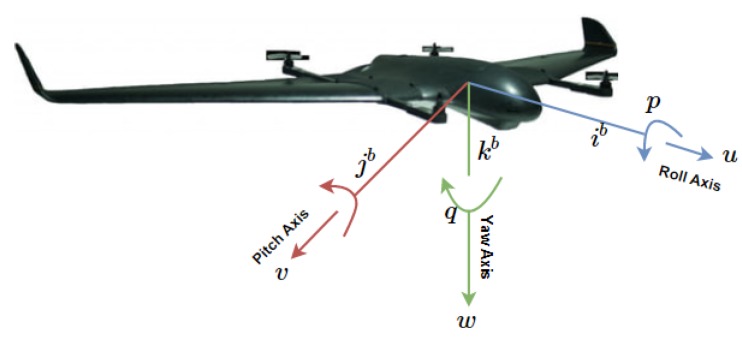
Body frame in a fixed-wing UAV.

Let us explain the modelling approach for fixed-wing UAVs, along the coordinate frame of Figure 11. For simplicity, let us remove the subscript *i* so as to avoid double indexing. Let us consider the states

(2) $$\begin{array}{c} q=\left[\begin{array}{c} X_{e}\\ E \end{array}\right],\;\dot{q}=\left[\begin{array}{c} V_{e}\\ \dot{E} \end{array}\right] \end{array}$$

which represent (angular) positions and (angular) velocities in the inertial frame. The last onese should not be confused with the (angular) velocities in the body frame, typically denoted with $\left[\begin{array}{c} V_{b}\\ \omega_{b} \end{array}\right]$. From basic mechanics, we get the following translational and rotational equations of motion

(3) $$\begin{array}{c} m\dot{V_{b}}+m\left(\omega_{b}\times V_{b}\right)+G\left(E\right)=\left[\begin{array}{c} \tau_{1}\\ \tau_{2}\\ \tau_{3} \end{array}\right]\\ I{\dot{\omega }}_{b}+\omega_{b}\times I\omega_{b}=\left[\begin{array}{c} \tau_{4}\\ \tau_{5}\\ \tau_{6} \end{array}\right] \end{array}$$

where *m* and *I* are the mass and the inertia matrix of the UAV. The relation between velocities represented in the inertial and the body frames can be expressed by

(4) $$\begin{array}{c} \dot{E}=J\omega_{b}\\ V_{e}=R_{b}^{e}V_{b} \end{array}$$

where *J* and $R_{b}^{e}$ are appropriate (rotation) matrices. Taking derivative of Equation (4), we obtain

(5) $$\begin{array}{c} \ddot{E}=J{\dot{\omega }}_{b}+\dot{J}\omega_{b}=J{\dot{\omega }}_{b}+\dot{J}J^{-1}\dot{E}\\ \dot{V_{e}}={\dot{R}}_{b}^{e}V_{b}+R_{b}^{e}{\dot{V}}_{b}={\dot{R}}_{b}^{e}R_{e}^{b}V_{e}+R_{b}^{e}{\dot{V}}_{b} \end{array}$$

which can be rearranged as

(6) $$\begin{array}{c} {\dot{\omega }}_{b}=J^{-1}\ddot{E}-J^{-1}\dot{J}J^{-1}\dot{E}\\ {\dot{V}}_{b}=R_{e}^{b}\dot{V_{e}}-R_{e}^{b}{\dot{R}}_{b}^{e}R_{e}^{b}V_{e} \end{array}$$

We can now substitute Equations (4) and (6) in the equations of motions (3), so as to obtain

(7) $$\begin{array}{c} m\left(R_{e}^{b}\dot{V_{e}}-R_{e}^{b}{\dot{R}}_{b}^{e}R_{e}^{b}V_{e}\right)+m\left(J^{-1}\dot{E}\times R_{e}^{b}V_{e}\right)+G\left(E\right)=\left[\begin{array}{c} \tau_{1}\\ \tau_{2}\\ \tau_{3} \end{array}\right]\\ I\left(J^{-1}\ddot{E}-J^{-1}\dot{J}J^{-1}\dot{E}\right)+\left(J^{-1}\dot{E}\right)\times I\left(J^{-1}\dot{E}\right)=\left[\begin{array}{c} \tau_{4}\\ \tau_{5}\\ \tau_{6} \end{array}\right] \end{array}$$

(8) $$\begin{array}{c} m\left(R_{e}^{b}\dot{V_{e}}\right)+m\left(J^{-1}\dot{E}\times R_{e}^{b}-R_{e}^{b}{\dot{R}}_{b}^{e}R_{e}^{b}\right)V_{e}+G\left(E\right)=\left[\begin{array}{c} \tau_{1}\\ \tau_{2}\\ \tau_{3} \end{array}\right]\\ I\left(J^{-1}\ddot{E}\right)+\left(\left(J^{-1}\dot{E}\right)\times IJ^{-1}-IJ^{-1}\dot{J}J^{-1}\right)\dot{E}=\left[\begin{array}{c} \tau_{4}\\ \tau_{5}\\ \tau_{6} \end{array}\right] \end{array}$$

and finally

(9) $$\begin{array}{c} m\left(R_{e}^{b}\dot{V_{e}}\right)+m\left(J^{-1}\dot{E}\times R_{e}^{b}-R_{e}^{b}{\dot{R}}_{b}^{e}R_{e}^{b}\right)V_{e}+G\left(E\right)=\left[\begin{array}{c} \tau_{1}\\ \tau_{2}\\ \tau_{3} \end{array}\right]\\ I\left(J^{-1}\ddot{E}\right)+\left(\left(J^{-1}\dot{E}\right)\times IJ^{-1}\dot{E}-IJ^{-1}\dot{J}J^{-1}\dot{E}\right)=\left[\begin{array}{c} \tau_{4}\\ \tau_{5}\\ \tau_{6} \end{array}\right] \end{array}$$

The expression (9) can be written in the Euler–Lagrange formalism as

(10) $$\begin{array}{c} \underset{D(q)}{\underbrace{\left[\begin{array}{cc} mR_{e}^{b} & 0\\ 0 & IJ^{-1} \end{array}\right]}}\underset{\ddot{q}}{\underbrace{\left[\begin{array}{c} {\dot{V}}_{e}\\ \ddot{E} \end{array}\right]}}+\underset{C(q,\dot{q})}{\underbrace{\left[\begin{array}{cc} m(\left(J^{-1}\dot{E}\right)\times R_{e}^{b}-R_{e}^{b}{\dot{R}}_{b}^{e}R_{e}^{b}) & 0\\ 0 & \left(J^{-1}\dot{E}\right)\times IJ^{-1}-IJ^{-1}\dot{J}J^{-1} \end{array}\right]}}\underset{\dot{q}}{\underbrace{\left[\begin{array}{c} V_{e}\\ \dot{E} \end{array}\right]}}+\underset{g(q)}{\underbrace{\left[\begin{array}{c} G(E)\\ 0 \end{array}\right]}}=\underset{\tau }{\underbrace{\left[\begin{array}{c} \tau_{1}\\ \tau_{2}\\ \tau_{3}\\ \tau_{4}\\ \tau_{5}\\ \tau_{6} \end{array}\right]}} \end{array}$$

where the different matrices are

(11) $$R_{e}^{b}\left(\phi ,\theta ,\psi \right)=\left[\begin{array}{ccc} c(\psi )c(\theta ) & c(\theta )s(\psi ) & -s(\theta )\\ c(\psi )s(\phi )s(\theta )-c(\phi )s(\psi ) & c(\phi )c(\psi )+s(\phi )s(\psi )s(\theta ) & c(\theta )s(\phi )\\ s(\phi )s(\psi )+c(\phi )c(\psi )s(\theta ) & c(\phi )s(\psi )s(\theta )-c(\psi )s(\phi ) & c(\phi )c(\theta ) \end{array}\right]$$

(12) $$D=\left[\begin{array}{cccccc} mc\left(\psi \right)c\left(\theta \right) & mc\left(\theta \right)s\left(\psi \right) & -ms\left(\theta \right) & 0 & 0 & 0\\ mc\left(\psi \right)s\left(\varphi \right)s\left(\theta \right)-mc\left(\varphi \right)s\left(\psi \right) & m\left(c\left(\varphi \right)c\left(\psi \right)+s\left(\varphi \right)s\left(\psi \right)s\left(\theta \right)\right) & mc\left(\theta \right)s\left(\varphi \right) & 0 & 0 & 0\\ m\left(s\left(\varphi \right)s\left(\psi \right)+c\left(\varphi \right)c\left(\psi \right)s\left(\theta \right)\right) & mc\left(\varphi \right)s\left(\psi \right)s\left(\theta \right)-mc\left(\psi \right)s\left(\varphi \right) & mc\left(\varphi \right)c\left(\theta \right) & 0 & 0 & 0\\ 0 & 0 & 0 & I_{x} & I_{\mathrm{xz}}s\left(\varphi \right) & -I_{x}s\left(\theta \right)-I_{\mathrm{xz}}c\left(\varphi \right)c\left(\theta \right)\\ 0 & 0 & 0 & 0\; & I_{y}c\left(\varphi \right) & I_{y}c\left(\theta \right)s\left(\varphi \right)\\ 0 & 0 & 0 & -I_{\mathrm{xz}} & -I_{z}s\left(\varphi \right) & I_{\mathrm{xz}}s\left(\theta \right)+I_{z}c\left(\varphi \right)c\left(\theta \right) \end{array}\right]$$

$$C=\left[\begin{array}{ccccc} 0 & 0 & 0 & 0 & 0\\ 0 & 0 & 0 & 0 & 0\\ 0 & 0 & 0 & 0 & 0\\ 0 & 0 & 0 & -\left(I_{\mathrm{xz}}-I_{x}s\left(\theta \right)\right)\dot{\theta } & I_{\mathrm{xz}}s\left(\varphi \right)s\left(\theta \right)\dot{\theta }-I_{z}s\left(\varphi \right)\dot{\theta }-I_{y}c\left(\varphi \right)s\left(\theta \right)\dot{\varphi }-I_{y}c\left(\varphi \right)\dot{\psi }\\ 0 & 0 & 0 & I_{x}\left(c\left(\varphi \right)\dot{\psi }-c\left(\theta \right)s\left(\varphi \right)\dot{\theta }\right)+I_{\mathrm{xz}}c\left(\varphi \right)\dot{\varphi } & s\left(\varphi \right)\left(I_{z}c\left(\varphi \right)\dot{\varphi }-\dot{\varphi }+I_{\mathrm{xz}}c\left(\varphi \right)\dot{\psi }-I_{\mathrm{xz}}c\left(\theta \right)s\left(\varphi \right)\dot{\theta }+I_{y}c\left(\varphi \right)c\left(\theta \right)\dot{\varphi }\right)\\ 0 & 0 & 0 & -I_{x}\left(s\left(\varphi \right)\dot{\psi }+c\left(\varphi \right)c\left(\theta \right)\dot{\theta }\right)-I_{\mathrm{xz}}s\left(\varphi \right)\dot{\varphi } & I_{y}{c\left(\varphi \right)}^{2}c\left(\theta \right)\dot{\varphi }-I_{z}{s\left(\varphi \right)}^{2}\dot{\varphi }-I_{\mathrm{xz}}{s\left(\varphi \right)}^{2}\dot{\psi }-c\left(\varphi \right)\dot{\varphi }-I_{\mathrm{xz}}c\left(\varphi \right)c\left(\theta \right)s\left(\varphi \right)\dot{\theta } \end{array}\right.\;\;\;$$

(13) $$\left.\begin{array}{c} 0\\ 0\\ 0\\ I_{x}{c\left(\theta \right)}^{2}\dot{\theta }-c\left(\theta \right)\dot{\theta }-I_{x}\dot{\theta }+I_{\mathrm{xz}}s\left(\theta \right)\dot{\theta }-I_{y}c\left(\theta \right)s\left(\varphi \right)\dot{\psi }+I_{z}c\left(\varphi \right)c\left(\theta \right)\dot{\theta }-I_{\mathrm{xz}}c\left(\varphi \right)c\left(\theta \right)s\left(\theta \right)\dot{\theta }-I_{y}c\left(\theta \right)s\left(\varphi \right)s\left(\theta \right)\dot{\varphi }\\ I_{y}{c\left(\theta \right)}^{2}\dot{\varphi }+c\left(\varphi \right)c\left(\theta \right)\dot{\varphi }-s\left(\varphi \right)s\left(\theta \right)\dot{\theta }-I_{\mathrm{xz}}c\left(\varphi \right)s\left(\theta \right)\dot{\varphi }-I_{x}c\left(\varphi \right)s\left(\theta \right)\dot{\psi }-I_{z}{c\left(\varphi \right)}^{2}c\left(\theta \right)\dot{\varphi }-I_{\mathrm{xz}}{c\left(\varphi \right)}^{2}c\left(\theta \right)\dot{\psi }-I_{y}{c\left(\varphi \right)}^{2}{c\left(\theta \right)}^{2}\dot{\varphi }+I_{x}c\left(\theta \right)s\left(\varphi \right)s\left(\theta \right)\dot{\theta }+I_{\mathrm{xz}}c\left(\varphi \right){c\left(\theta \right)}^{2}s\left(\varphi \right)\dot{\theta }\\ I_{\mathrm{xz}}s\left(\varphi \right)s\left(\theta \right)\dot{\varphi }-c\left(\varphi \right)s\left(\theta \right)\dot{\theta }-c\left(\theta \right)s\left(\varphi \right)\dot{\varphi }+I_{x}s\left(\varphi \right)s\left(\theta \right)\dot{\psi }+I_{\mathrm{xz}}{c\left(\varphi \right)}^{2}{c\left(\theta \right)}^{2}\dot{\theta }+I_{z}c\left(\varphi \right)c\left(\theta \right)s\left(\varphi \right)\dot{\varphi }+I_{\mathrm{xz}}c\left(\varphi \right)c\left(\theta \right)s\left(\varphi \right)\dot{\psi }+I_{x}c\left(\varphi \right)c\left(\theta \right)s\left(\theta \right)\dot{\theta }+I_{y}c\left(\varphi \right){c\left(\theta \right)}^{2}s\left(\varphi \right)\dot{\varphi } \end{array}\right]$$

(14) $$G=\left[\begin{array}{c} -g_{a}ms\left(\theta \right)\\ g_{a}mc\left(\theta \right)s\left(\varphi \right)\\ g_{a}mc\left(\varphi \right)c\left(\theta \right) \end{array}\right],\;J=\left[\begin{array}{ccc} 1 & s(\phi )s(\theta )/c(\theta ) & c(\phi )s(\theta )/c(\theta )\\ 0 & c(\phi ) & -s(\phi )\\ 0 & s(\phi )/c(\theta ) & c(\psi )/c(\theta ) \end{array}\right]$$

and the notation $c\left(\cdot \right)$, $s\left(\cdot \right)$ has been used as an abbreviation of $\cos\left(\cdot \right)$, $\sin\left(\cdot \right)$.

All the aforementioned UAV dynamics have been implemented in C++ via ’Odeint’, which is a C++ library for numerically solving ordinary differential equations [37]. The library provides various solvers like Runge–Kutta4, Dormand–Prince, etc. The Odeint solvers need a functor or a function describing the UAV dynamics. Thus, the dynamics of the body can be simulated in C++ and eventually run on a machine or on Raspberry Pi as a “virtual drone”. Alternatively, one can rely on Gazebo to simulate the drone dynamics.

## 5. Control Architecture

**Figure 12:**
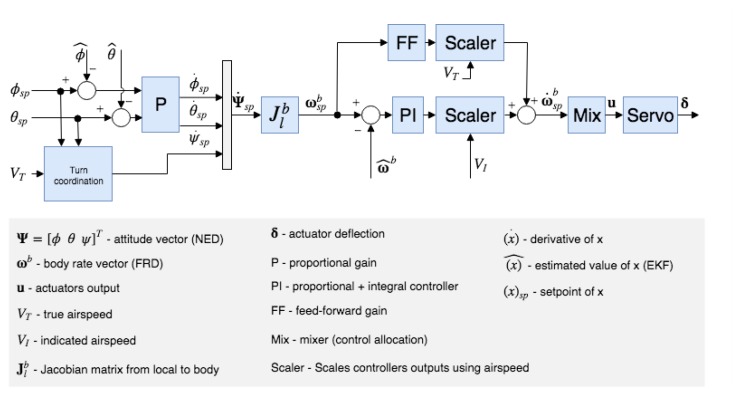
Attitude control loop for fixed-wing UAVs in PX4.

The PX4 stack realizes a discrete time cascaded control loop for attitude control as shown in the Figure 12. The outer loop is a Proportional (P) controller. This controller operates on the error between the setpoint and the estimated attitude to generate a rate setpoint. The inner Proportional-Integral (PI) controller uses the error in rates to compute the required angular acceleration. The autopilot layer is completed with Feed-Forward (FF) and scaling terms that must be typically tuned to improve performance. Standard autopilots for fixed-wing UAVs realize at least the following four low-level controllers [38]

1. Yaw rate control,
2. Pitch angle control,
3. Roll angle control,
4. Total Energy Control System (TECS) for height and velocity control.

The controllers are realized as discrete time control loops. With such an architecture, the autopilot software issues commands to the interfaced actuators for the required angular acceleration.

On top of the autopilot software is a path-following algorithm which can be of the type in [15,16] (vector-field, carrot-chasing, nonlinear guidance, pure pursuit with line-of-sight, linear quadratic regulation), and thus is not presented for compactness.

Rather than presenting a path-following algorithm and augment it with a formation control module, let us follow a more concise presentation due to space limitations and due to intellectual property rights of the integrated path-following/formation-control module. We will present an example of a formation control algorithm for Euler–Lagrange systems in the form (10) that can be integrated with a path-following algorithm.

We consider networks of Euler–Lagrange agent which are linked to each other via a communication graph that describes the allowed information flow. A directed graph or digraph is composed of nodes and directed edges (arrows). A directed graph can be written as an ordered pair,

(15) D=(V,A)

where, V is a set of node (or vertices), and A is a set of ordered pair of nodes called arrows. A graph representation is useful since each UAV forms a node in the graph and the directed edges represent the allowed information flows between the UAVs. For graph based approach of formation control, nodes in the graph can be classified into three based on information flow as,

- Path planner node: This node decides the path for the complete set of drones. For example, the path can be determined according to a vector-field algorithm. The node does not receive information from any other nodes (UAVs) and also generates the dynamics to which all other nodes should synchronize. Thus the node is called as pinner node in literature.
- Leader node: Leader nodes in the formation have access to data from the pinner node.
- Follower nodes: The follower nodes have only access to data from nodes other than the pinner node.

**Figure 13:**
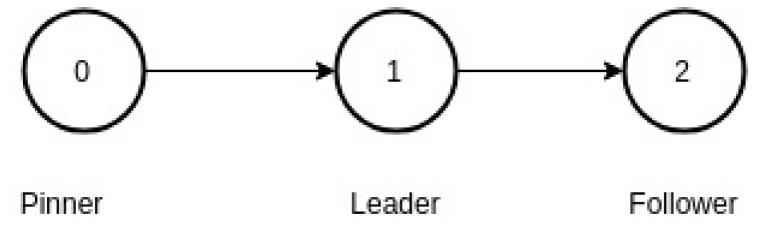
A simple communication graph with path planner, leader and follower.

An example of a network of UAVs is in the communication graph depicted in Figure 13. Node 0 is the pinner node; node 1, the leader node, receives information from the pinner node but not vice versa. Node 2, the follower node, has access to the information from Node 1. Mathematically the communication graph for Figure 13 can be written as D=(V,A), where V={0,1,2}, and A={(0,1),(1,2)}.

**Figure 14:**
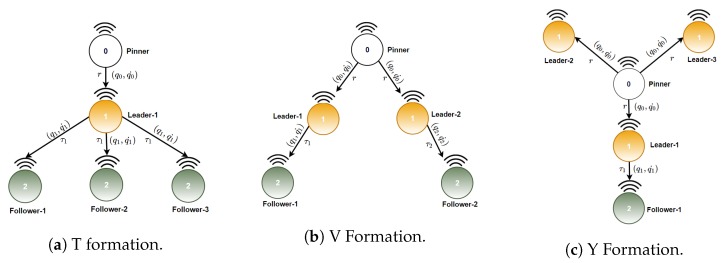
Communication graphs for inverted T, inverted V and Y formations.

The graphs corresponding to inverted T, inverted V, and Y formations are shown in Figure 14: Such formations will be studied in this work. The path planner is indicated with white color, leaders are indicate with orange colors, and followers with green colors. It is worth noticing that each formation has a different number of leaders and followers: This implies that switching formation require to rearrange the communication and the control layers.

### 5.1. Reference Dynamics for Leader/Follower Synchronization

The graph based Leader/Follower formation control approach aims to by synchronizing to given reference dynamics [39,40]. The reference dynamics for this is formulated as,

(16) $$\left[\begin{array}{c} {\dot{q}}_{0}\\ {\ddot{q}}_{0} \end{array}\right]=\underset{A_{m}}{\underbrace{\left[\begin{array}{cc} 0 & 𝟙\\ -K_{p} & -K_{v} \end{array}\right]}}\underset{x_{m}}{\underbrace{\left[\begin{array}{c} q_{0}\\ {\dot{q}}_{0} \end{array}\right]}}+\underset{B_{m}}{\underbrace{\left[\begin{array}{c} 0\\ 𝟙 \end{array}\right]}}r$$

where $q_{0},{\dot{q}}_{0}\in R^{n}$, $x_{m}$ the reference model states, $K_{p}$, $K_{v}$ are the proportional and derivative gains of the multivariable PD controller, 𝟙 indicated an identity matrix of appropriate dimension, and $r={\ddot{q}}^{d}+K_{v}{\dot{q}}^{d}+K_{p}q^{d}$ is a control input.

Currently, the control in path planning UAV has a vector field based approach which does not ensure a dynamics as given in (16) for the UAV. Thus, the path planner node also needs to generate a reference dynamics to which all UAVs in the formation should synchronize. On using an inverse dynamic based controller of the form in (17) we obtain the dynamics as in (16).

(17) $$\tau =D\left(q\right)a+C\left(q,\dot{q}\right)\dot{q}+g\left(q\right)$$

where the term *a* is defined as

(18) $$a={\ddot{q}}^{d}-K_{v}\dot{e}-K_{p}e$$

with $e=q-q^{d}$. We obtain the error dynamics as,

(19) $$\ddot{e}+K_{v}\dot{e}+K_{p}e=0$$

The equation in (19) can be re-written in state space form as,

(20) $$\left[\begin{array}{c} \dot{e}\\ \ddot{e} \end{array}\right]=\left[\begin{array}{cc} 0 & 𝟙\\ -K_{p} & -K_{v} \end{array}\right]\left[\begin{array}{c} e\\ \dot{e} \end{array}\right]$$

Since $K_{p}$, $K_{v}$ are positive gains, by construction the state matrix in (20) is Hurwitz. This implies as t→∞, e→0 [41] i.e., $q\to q_{d}$. Moreover, (20) can be easily re-written to obtain the form in (16).

In path planner node, we run the dynamics in (16) virtually with $q^{d}$, ${\dot{q}}^{d}$,and ${\ddot{q}}^{d}$ as the inertial measurements (trajectories, velocities, and accelerations) of the path planner UAV. By this method, the reference states $x_{m}$ will be in close match to the states of the path planner UAV and have the dynamics in (16). The reference states $x_{m}$ are further transmitted to the leader nodes for leader synchronization.

### 5.2. Synchronization of Leader Dynamics to Reference Dynamics

The state–space representation of EL dynamics for any UAV is

(21) $$\underset{{\dot{x}}_{l}}{\underbrace{\left[\begin{array}{c} {\dot{q}}_{l}\\ {\ddot{q}}_{l} \end{array}\right]}}=\underset{A_{l}}{\underbrace{\left[\begin{array}{cc} 0 & 𝟙\\ 0 & -D_{l}^{-1}C_{l} \end{array}\right]}}\underset{x_{l}}{\underbrace{\left[\begin{array}{c} q_{l}\\ {\dot{q}}_{l} \end{array}\right]}}+\left[\begin{array}{c} 0\\ -D_{l}^{-1}g_{l} \end{array}\right]+\underset{B_{l}}{\underbrace{\left[\begin{array}{c} 0\\ D_{l}^{-1} \end{array}\right]}}\tau_{l}$$

In this section, the subscript *l* in (21) represents the values for leader type UAVs. Model reference control [42] is a typical control approach in which the plant is made to have the dynamics as of a reference model by using an appropriate control law. The control law is developed by first defining a control structure, and then finding matching conditions that makes the closed loop dynamics as that of the reference model. The ideal model reference control law for this purpose would be

(22) $$\tau_{l}^{*}=D_{l}\left(-K_{p}q_{l}-K_{v}{\dot{q}}_{l}+r\right)+C_{l}{\dot{q}}_{l}+g_{l}$$

where $r={\ddot{q}}^{d}+K_{v}{\dot{q}}^{d}+K_{p}q^{d}$ is a control input used in the path planner node.

### 5.3. Synchronization of Follower Dynamics to Reference Dynamics

The follower synchronizes to reference dynamics exploiting the signals of the neighbouring agents. Here we consider only followers which listen to the data from one neighbour. The state–space representation of EL dynamics for a follower node is

(23) $$\underset{{\dot{x}}_{f}}{\underbrace{\left[\begin{array}{c} {\dot{q}}_{f}\\ {\ddot{q}}_{f} \end{array}\right]}}=\underset{A_{f}}{\underbrace{\left[\begin{array}{cc} 0 & 𝟙\\ 0 & -D_{f}^{-1}C_{f} \end{array}\right]}}\underset{x_{f}}{\underbrace{\left[\begin{array}{c} q_{f}\\ {\dot{q}}_{f} \end{array}\right]}}+\left[\begin{array}{c} 0\\ -D_{f}^{-1}g_{f} \end{array}\right]+\underset{B_{f}}{\underbrace{\left[\begin{array}{c} 0\\ D_{f}^{-1} \end{array}\right]}}\tau_{f}$$

Similarly, the dynamics of any neighbouring agent can be written as,

(24) $$\underset{{\dot{x}}_{n}}{\underbrace{\left[\begin{array}{c} {\dot{q}}_{n}\\ {\ddot{q}}_{n} \end{array}\right]}}=\underset{A_{n}}{\underbrace{\left[\begin{array}{cc} 0 & 𝟙\\ 0 & -D_{n}^{-1}C_{n} \end{array}\right]}}\underset{x_{n}}{\underbrace{\left[\begin{array}{c} q_{n}\\ {\dot{q}}_{n} \end{array}\right]}}+\left[\begin{array}{c} 0\\ -D_{n}^{-1}g_{n} \end{array}\right]+\underset{B_{n}}{\underbrace{\left[\begin{array}{c} 0\\ D_{n}^{-1} \end{array}\right]}}\tau_{n}$$

The model reference control law for any follower is

(25) $$\begin{array}{c} \tau_{f}^{*}=C_{f}{\dot{q}}_{f}+D_{f}D_{n}^{-1}\tau_{n}-D_{f}D_{n}^{-1}C_{n}{\dot{q}}_{n}-D_{f}D_{n}^{-1}g_{n}-D_{f}\left(K_{p}{\bar{e}}_{fn}+K_{v}{\bar{\bar{e}}}_{fn}\right)+g_{f} \end{array}$$

where ${\bar{e}}_{fn}=q_{f}-q_{n}$, and ${\bar{\bar{e}}}_{fn}={\dot{q}}_{f}-{\dot{q}}_{n}$. With the proposed architecture, it is also possible to enable topology to switch during flight. The idea is to use a server/client setup where the UAVs in formation are connected to a server via wireless access point. The server program receives user input for specific formation and distributes formation gap data between the UAVs. On receiving these data, the Raspberry Pi executes the formation control algorithm and issues commands to the autopilot using onboard APIs.

## 6. Results

Because it is difficult to provide clear plots in a Gazebo environment (see Figure 3 and Figure 4), in the following we will provide software-in-the-loop simulation results, visualized in a Matlab environment. For five UAVs all algorithms run in real-time in the machine where the software for PX4 and Raspberry Pi has been uploaded. First, results for take-off and loitering of fixed-wing UAVs are shown.

**Figure 15:**
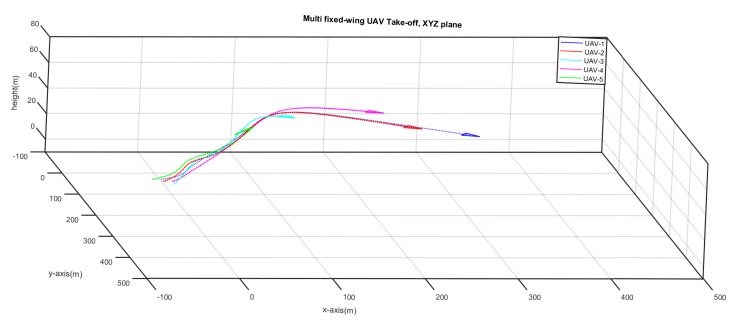
UAVS taking-off from different positions at different time instants.

**Figure 16:**
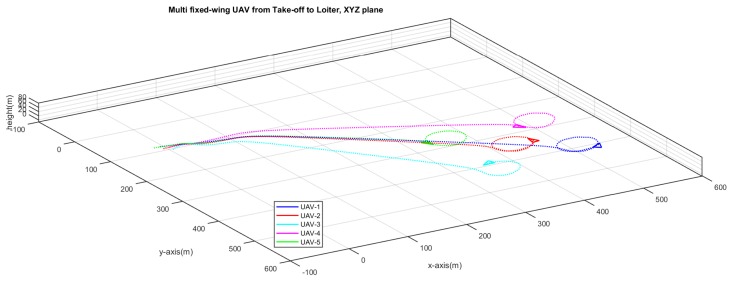
After take-off, the UAVs reach different loitering points.

Figure 15 shows the that five UAVs have took off from different positions on the ground and they are following a trajectory at different time instant. Figure 16 shows that the UAVs have followed their trajectories and are now loitering around their respective points. No formation control is enabled yet.

**Figure 17:**
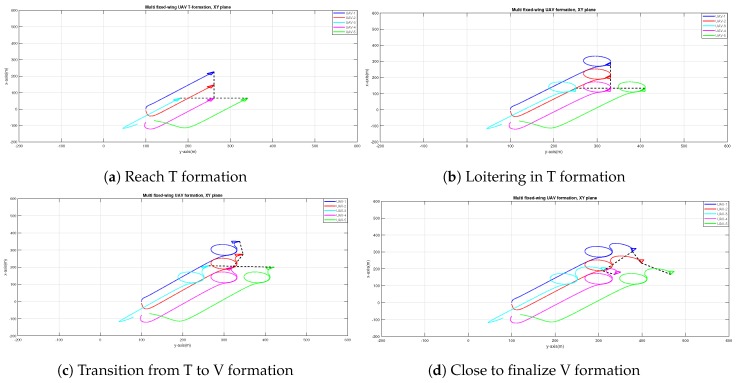
Different stages of the transition from inverted T to inverted V formation.

It must be further underlined that the software implementation of each UAV node has been logically organized by splitting a given mission into straight line and orbit primitives: Way points are provided to the leader formation and the mission is automatically split into lines connecting the way points and orbit loitering around the way points. Furthermore, commands can be given in real-time to reconfigure the formation from one shape to a different one. To highlight the capabilities of switching formation, let us first look at Figure 17a,b, where the starting point is an inverted T formation. Upon reconfiguring the formation gaps, the formation can transit from inverted T to inverted V, as depicted in Figure 17c,d. The trails from the previous formation (T-formation) are removed to keep the picture neat.

**Figure 18:**
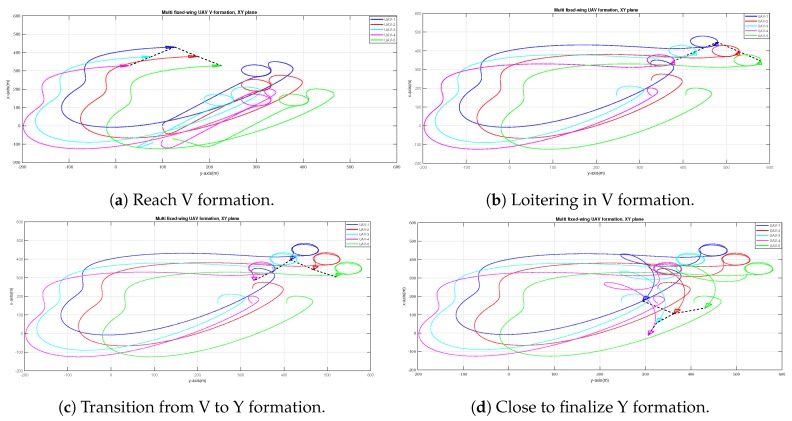
Different stages of the transition from inverted Y to Y formation.

Figure 18a,b complete the transition along three formations: After going from inverted T to inverted V formation, the UAVs go from inverted V to Y formation (Figure 18c,d).

**Figure 19:**
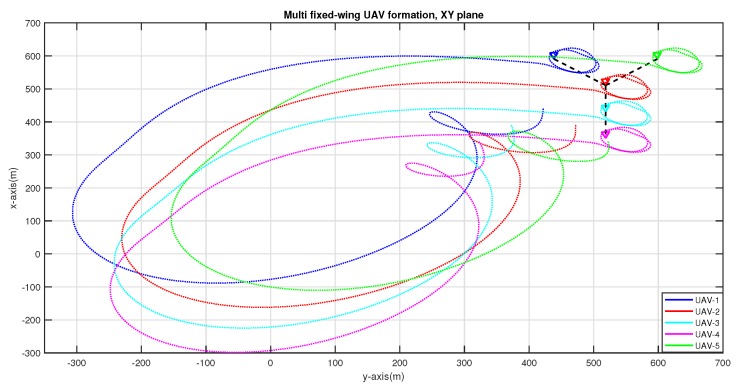
UAVs have completed the transition from V to Y formation and start loitering.

Finally, the simulation ends with Figure 19 showing the flock reaching at the loitering point in Y formation. As it can be seen trails of previous formation (T and V formations) are removed to keep the picture neat. At this point formation of all three formations while UAVs being airborne is shown.

Overall, the proposed environment shows a validation of guidance and formation control algorithms in the presence of low-level control performance, communication protocols and unreliable communication.

## 7. Conclusions

Despite the ongoing research on the topics of guidance and formation control of fixed-wing Unmanned Aerial Vehicles (UAVs), little progress is known on implementation of semi-physical validation platforms (hardware-in-the-loop or software-in-the-loop) of such complex systems. This paper describes the development of a semi-physical platform for multi-fixed wing UAVs where not only the physical aspects of UAV dynamics are captured, but also the cybernetics aspects such as the autopilot and the communication layers connecting the different components. The environment adopts Raspberry Pi’s programmed in C++, which can be interfaced to standard autopilots (PX4) as a companion computer. Simulations are done in a distributed setting with a server program designed for the purpose of handling the user inputs and configurations of the UAVs. Gazebo-ROS is used as a 3D visualization tool.

This work opens up many possible research directions. To start with, the development and integration of adaptive guidance and formation algorithm utilizing Gazebo as simulator: Adaptive guidance algorithms could be designed to handle uncertainty in course dynamics and uncertainty in UAV environments. Second, the platforms opens the possibility of studying long-distance protocol for communication and ad-hoc networking (Zigbee, Ad-hoc Wi-Fi, LoRaWAN, etc.) to set up communications during formation control. The new packet protocol introduced for inter-UAV communication is written above the transport layer, and can be easily made to use any of the above technologies. Moreover, an interesting topic is how to minimize communication in such a way to minimize the chance of packet losses. Another research direction comes from the fact that hardware-in-the-loop simulations performed in this work exhibited communication overhead due to the UAV physics model running in the Gazebo simulator: The technique of simulation-in-hardware (running the UAV physics model in Raspberry Pi) can be performed to achieve lower communication overhead and increase the real-time performance of the simulator.
